# Synergistic effects of rhizosphere effect and combined organic and chemical fertilizers application on soil bacterial diversity and community structure in oilseed rape cultivation

**DOI:** 10.3389/fmicb.2024.1374199

**Published:** 2024-03-14

**Authors:** Jingyuan Wang, Hongling Qin, Leyan Zhang, Yafang Tang, Junjiang Long, Huaqin Xu, Baoli Zhu

**Affiliations:** ^1^College of Environment and Ecology of Hunan Agricultural University, Changsha, China; ^2^Key Laboratory of Agro-ecological Processes in Subtropical Region, Institute of Subtropical Agriculture, Chinese Academy of Sciences, Changsha, China; ^3^Hubei Key Laboratory of Quality Control of Characteristic Fruits and Vegetables, College of Life Science and Technology, Hubei Engineering University, Xiaogan, China

**Keywords:** oilseed rape, organic fertilizer, chemical fertilizer, rhizosphere effect, root exudates

## Abstract

The combined application of chemical and organic fertilizers has been recognized to enhance soil fertility and foster the soil microbial ecosystem. However, the optimal ratio of chemical and organic fertilizers in oilseed rape cultivation is still uncertain, and the role of rhizosphere effect is still unclear. Thus, this study aimed to elucidate the impacts of varying ratios of chemical and organic fertilizers on the structure and potential functionalities of rhizosphere and non-rhizosphere soil microbial communities. The interplay of microbial communities with soil properties and oilseed rape root exudates was investigated in controlled pot cultivations receiving varying ratios of chemical and organic fertilizers. Results indicated clear segregation in the soil bacterial community, influenced by both fertilization treatments and rhizosphere effects. The bacterial community structure significantly correlated with nitrate nitrogen, organic acids, and dissolved organic carbon (DOC) content. Rhizosphere effects led to increased bacteria abundance, reduced diversity, and decreased network stability. Notably, F3 treatment receiving 25% chemical and 75% organic fertilizers showed a significantly higher abundance at 1.43 × 10^11^ copies g^−1^ dry soil, accompanied by increased species and genetic diversity, and ecological network complexity. This treatment also yielded the highest aboveground biomass of oilseed rape. However, the application of organic fertilizers also increased the risk of plant pathogenicity. This study reveals the impact of fertilizers and rhizosphere effects on soil microbial community structure and function, shedding light on the establishment of more effective fertilization schemes for oilseed rape agriculture.

## 1 Introduction

In agriculture, the augmentation of soil fertility usually involves substantial fertilizer applications to bolster crop yields (Brtnicky et al., 2023). However, the excessive reliance on chemical fertilizers has led to a range of environmental concerns, including biodiversity loss, soil acidification and degradation, greenhouse emissions, and contamination of ground and surface water systems (Searchinger et al., 2018; Yu et al., 2020; Kanthilanka et al., 2023). To mitigate the adverse repercussions of chemical fertilizer application, organic fertilizers have emerged as a potential alternative strategy (Chen et al., 2020; Kumari et al., 2024). Numerous studies have underscored the capacity of organic fertilizers to improve soil fertility and promote crop growth (Cui et al., 2018; Maltas et al., 2018; Wu et al., 2018; He et al., 2022; Li et al., 2024). However, organic fertilizer also has limitations, such as the nutrient release rate is often slow (Garzón et al., 2011; Zhang et al., 2024). Combined application of organic and chemical fertilizers has positive effects on soil fertility and microbial properties compared to application of single type of fertilizers (Qaswar et al., 2020; Kumari et al., 2024). Studies suggest prolonged application of mixed organic and chemical fertilizers improves crop growth, nutrient utilization, and yield stability across various crops, including wheat, apple, rice, and other crops (Wang et al., 2023; Zhao et al., 2023; Lu et al., 2024). This practice also nurtures bacterial resilience to environmental changes, promoting soil carbon and nitrogen cycling (Chen W. et al., 2023). Fertilizer application strategy and rates affect soil fertility, microorganisms, and crop growth (Li D. et al., 2023), reducing the amount of chemical fertilizers and determining the optimal ratio of organic fertilizers to chemical fertilizers are key measures to achieve sustainable agricultural development.

Soil microbial communities are integral to an array of soil- and plant-related processes and play a pivotal role in regulating plant health (Fahey et al., 2020). Microbial communities are fundamental in maintaining agricultural sustainability and the overall functioning of soil ecosystems (Trivedi et al., 2016; Manoharan et al., 2017; Vezzani et al., 2018). In recent years, the rhizosphere microbiota has received much attention due to the important role of beneficial interactions between roots and rhizosphere microorganisms in fostering plant growth (Zheng et al., 2017). Notably, rhizosphere microorganisms are regarded as the second genome of plants (Deng et al., 2021). These microorganisms can enhance nutrient availability, promote plant growth, and confer resilience on plants against various abiotic and biotic stresses (Zheng et al., 2018; Ali and Khan, 2021). Recent evidence suggests that an increase in endospheric microorganisms diversity and abundance under stress conditions might contribute to plant survival, suggesting a significant role of microbes in improving plant health (Yuan et al., 2018).

Soil microorganisms are sensitive to environmental changes, and fertilization often exerts a significant influence on microbial activity (Francioli et al., 2016). Studies have shown that moderate organic fertilizer applications increase bacterial abundance and diversity (Liu et al., 2021, 2023). For example, rhizosphere microbial biomass increased and endosphere microbial community composition changed significantly after the application of organic fertilizer (Peng et al., 2016); the combination of organic fertilizer and chemical fertilizer improved the soil quality of maize arable land, increased the diversity of bacterial communities, and led to a more stable bacterial network and an increase in the complexity of potential microbial metabolites (Lu et al., 2024). In addition, bacterial community composition varies depending on the proportion of organic fertilizer applied. For example, manure treatment enriched bacterial taxa involved in phosphorus transformation (Zhang et al., 2023). Thus, the blending of organic and chemical fertilizers emerges as a promising avenue to regulate soil health and enhance the microecological environment by modulating the soil bacterial community. However, there remains a dearth of research on the effects of organic-chemical fertilizer combinations on rhizosphere processes mediated by microbes and the intricate relationship between microbial diversity, soil properties, and crop root systems, particularly under diverse fertilization conditions (Pang et al., 2021).

Oilseed rape is a major cash crop in China and serves as a vital feedstock for biofuel production. While previous studies have demonstrated significant soil quality and yield improvements with the combined application of organic and chemical fertilizers, the specific impacts of chemical-organic fertilizer dosing on soil microbiology in oilseed rape cultivation remain nebulous. Therefore, this study aims to investigate the effects of chemical and organic fertilizer allocation alongside rhizosphere effects on the structure and potential functionalities of microbial communities in oilseed rape soils, and to determine the optimal chemical and organic fertilizer ratio for reducing chemical fertilizer usage while promoting oilseed rape growth, laying a theoretical foundation for the eco-efficient management of oilseed rape cultivation.

## 2 Materials and methods

### 2.1 Test materials and site selection

The experiment was conducted in a well-lit and ventilated greenhouse at the Institute of Subtropical Agriculture, Chinese Academy of Sciences (118°05′E, 28°12’N). The oilseed rape cultivar was Xiangmai Oil No. 6. The test soil was collected from 0 to 20 cm of dryland soil at the Taoyuan Agricultural Ecology Experimental Station of the Chinese Academy of Sciences (111°26′E, 28°55’N, 92.2 m above sea level). The soil used was typical red soil in subtropical regions (derived from Quaternary red clay), with a pH of 4.5, organic matter of 13.81 g kg^−1^, total nitrogen 0.84 g kg^−1^, total phosphorus 0.38 g kg^−1^, total potassium 12.33 g kg^−1^, alkaline dissolved nitrogen 77.81 g kg^−1^, effective phosphorus 6.05 g kg^−1^, and quick-acting potassium 103.40 g kg^−1^.

### 2.2 Experimental design

The soil used for cultivation was air-dried and crushed, then passed through a 2-mm sieve, and after removing residues and impurities, the soil was mixed with fertilizers of different organic and chemical fertilizer ratios. Five fertilization treatments were set up, each receiving the same amount of total N, i.e., F0: application of chemical fertilizer alone (CK) was used as the control; F1: 25% organic fertilizer and 75% chemical fertilizer (1:3); F2: 50% organic fertilizer and 50% chemical fertilizer (1:1); F3: 75% organic fertilizer and 25% chemical fertilizer (3:1); and F4: only organic fertilizer was applied. Each treatment contained four replicates. Fertilizers with different ratios of chemical fertilizers were mixed well, considering the amount of fertilizers applied in the field (Nitrogen fertilizers: 105–225 kg N ha^−1^, Phosphorus fertilizers: 45–78 kg P ha^−1^, Potassium fertilizers: 78–137 kg K ha^−1^), so that the content of the fertilizers added to the potting soil eventually reached 200 mg kg^−1^ of N. Nitrogen, Phosphorus, and potassium fertilizers were added in the form of urea, calcium superphosphate, and potassium chloride, respectively, and organic fertilizers used were fermented and then air-dried cow dung, with nutrient contents of total N 1.09 g kg^−1^, total P (as P_2_O_5_) 4.39 g kg^−1^ and total K (as K_2_O) 18.46 g kg^−1^.

PVC pots, 20 cm high and 16 cm in diameter, were used for cultivation experiments. Each pot was filled with 3.5 kg of mixed fertilizer soil, and the water content was adjusted to 75% of the soil water holding capacity. Then, three 2-week-old rapeseed seedlings in uniform growth were transplanted. After 15 days, the seedlings were planted. On day 58, when the rapeseed reached the budding stage, samples of rhizosphere soil (collected using the root shaking method) and non-rhizosphere soil were obtained. A portion of these soil samples was preserved in a refrigerator at 4°C for subsequent analysis of parameters including ammonium nitrogen, nitrate nitrogen, water content, soluble organic carbon, and rhizosphere secretion. The remaining portion underwent quick-freezing with liquid nitrogen and was stored at −80°C for molecular analysis.

### 2.3 Determination of rhizosphere exudates

Root exudates were collected via *ex-situ* incubation in 5 mM calcium chloride for 6 h, and then the total amount of amino acids was determined by an automatic amino acid analyzer (Hitachi L8900). The total amount of organic acids in the rhizosphere soil was detected with high-performance liquid chromatography (HPLC).

### 2.4 DNA extraction and library construction for 16S rRNA amplicon sequencing

DNA was extracted by the CTAB method and DNA quality was determined using a NanoDrop 1,000 spectrophotometer (Thermo Fisher Scientific, Wilmington, United States). Bacterial abundance was then determined by qPCR using a Roche LightCycler480II fluorescence quantitative PCR instrument. The bacterial 16S rRNA gene was amplified by polymerase chain reaction (PCR) using primer pair 338F (5′-ACTCCTACGGGGAGGCAGCA3′) and 806R (5′-GGACTACHVGGGTWTCTAAT-3′) (Langille et al., 2013). The amplicon samples were sequenced at MajorbioBio-Pharm Technology Co. Ltd. (Shanghai, China) using the Illumina MiSeq platform. After removing low quality reads, remaining sequences were divided into OTUs at 97% similarity using Uparse (version 7.0.1090) and were analyzed for other bioinformatic statistics.

### 2.5 Data analysis

Soil physicochemical properties have been determined in a previous study and the results of this data were used (Wang et al., 2021). One-way analysis of variance (ANOVA) was performed using SPSS 26.0 to determine differences in microbial composition and microbial α-diversity between treatments. Correlations between environmental factors, bacterial abundance, and species α-diversity were then plotted using origin2022b, and correlation heatmaps were utilized to assess the correlation between environmental factors, bacterial abundance, and species α-diversity, and differences in means were considered statistically significant if *p* < 0.05. Non-metric multidimensional scaling (NMDS) was used to investigate the effect of treatments on microbial community composition at the microbial OTU level. To compare α-diversity among treatments, OTU data were analyzed using the phylogenetic diversity (PD) metric and Shannon index. Redundancy analysis (RDA) was performed by Canoco5 where the statistical methods used to assess the significance of environmental factors were Monte Carlo tests, and ANOSIM (analysis of similarity) based on the OTU’s Bray-Curtis distances was used to measure the effects of fertilizer application and rhizosphere effects on the composition of the bacterial community.

Full length 16S rRNA sequences of common pathogens were retrieved from NCBI, and were used as query to blast against our sequences using TBtools. Hit sequences with > = 94% identity were considered as the same genus of respective pathogen. To reveal the potential effects of fertilization practices on microbial interactions, OTUs with abundance greater than 0.5% were used to construct microbial networks with Spearman using the “psych” package in R (version 3.6.3), and were visualized with Gephi 0.9.2. and set the set the *p*-value threshold to 0.01 and the correlation coefficient threshold to 0.7 to filter out the relationships with less than a certain correlation or frequency of co-occurrence. Complexity of the network and highlight important relationships. and various topological features of the bacterial networks were computed to infer the network characteristics.

## 3 Results

### 3.1 Soil bacterial abundance

The abundance of rhizosphere bacteria significantly responded to fertilizer treatments (Table 1), demonstrating an escalating trend with higher proportions of organic fertilizers (Supplementary Figure S1). Notably, F3 exhibited the highest bacterial abundance at 1.43 × 10^11^ copies g^−1^ dry soil, while F1 had the lowest at 2.96 × 10^10^ copies g^−1^. In non-rhizosphere soils, the impact of fertilizer treatments on bacterial abundance was less pronounced, showing no significant differences among treatments. Interestingly, bacterial abundance in the F4 treatment receiving only chemical fertilizer was much higher in the rhizosphere soil than in the non-rhizosphere. Rhizosphere bacterial 16S rRNA gene abundance showed a positive correlation with total amino acids, while non-rhizosphere abundance correlated positively with soil water content (Supplementary Figure S2).

**Table 1:** Rhizosphere effects and fertilizer treatments on soil bacterial abundance and α-diversity.

Group	Abundance (16S rRNA)		Shannon index		Phylogenetic diversity	
	*F*	*p*-value	*F*	*p*-value	*F*	*p*-value
REs	8.728	0.006**	9.999	0.004**	5.563	0.025*
Fs	2.105	0.105	2.381	0.074	26.442	0.000**
REs*Fs	2.287	0.083	1.534	0.218	0.470	0.757

REs, Rhizosphere effects; Fs, Fertilization treatments; REs*Fs, Interaction of rhizosphere effect and fertilization treatments.

### 3.2 Soil microbial diversity

**Figure 1:**
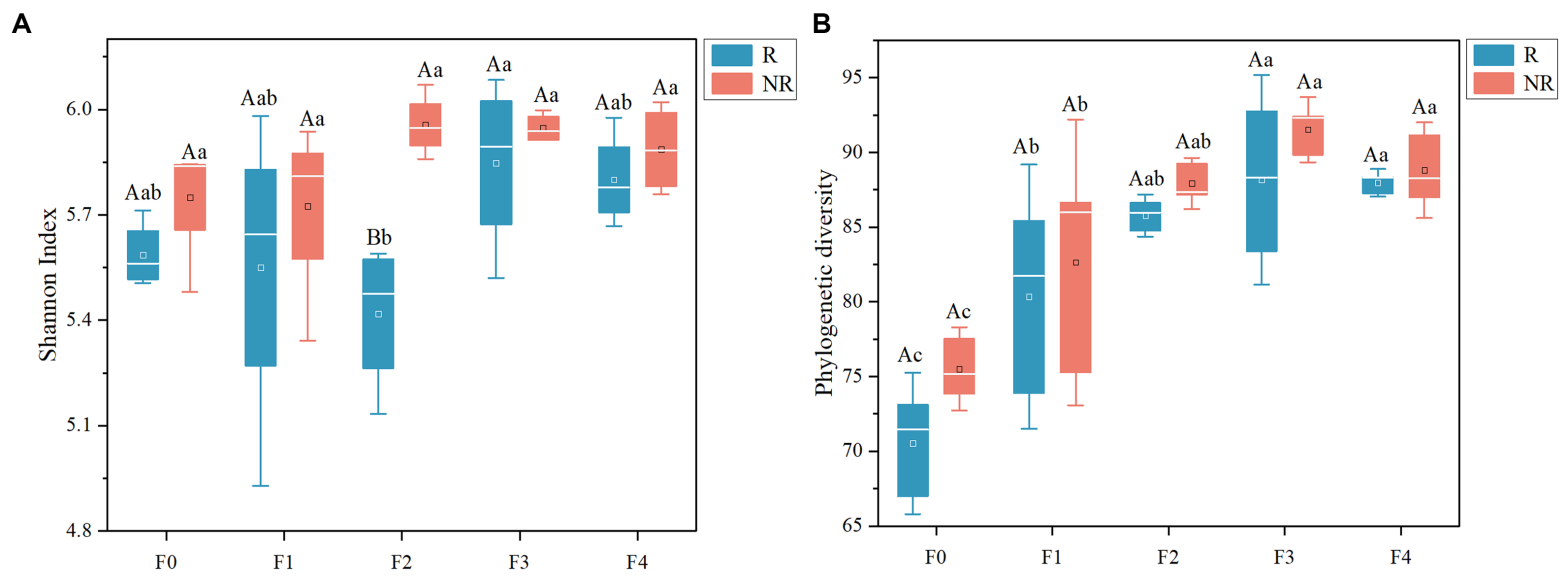
Phylogenetic diversity **(A)** and Shannon index **(B)** of bacterial communities in soil. Uppercase letters indicate differences between rhizosphere and non-rhizosphere between each group of fertilizer treatments; lowercase letters indicate differences between fertilizer treatments (*p* < 0.05).

A total of 2,240,027 high-quality sequences with an average length of 432 bp were obtained in this study, and a total of 3,092 operational taxonomic units (OTUs) were classified at 97% sequence similarity cutoff. Differences in microbial diversity existed between the rhizosphere and non-rhizosphere (Table 1). In the F2 treatment receiving equal proportion of organic and chemical fertilizers, the Shannon index of the rhizosphere microbes was lower than that of the non-rhizosphere soil (Figure 1A). Organic fertilizer enhanced microbial PD in both rhizosphere and bulk soils, even if it was applied at the minimum level (25%). An increasing proportion of organic fertilizer further increased the PD index, which remained relatively stable at all other treatments (Figure 1B). However, the impact of organic fertilizer application on the Shannon index was ambiguous (Figure 1A). These findings suggest that organic fertilizer sustains a higher microbial functional diversity. Further analysis indicated that the PD index positively correlated with amino acids content and DOC in rhizosphere soil and with nitrate-N in non-rhizosphere soil (Supplementary Figure S2).

### 3.3 Soil microbial community composition

**Figure 2:**
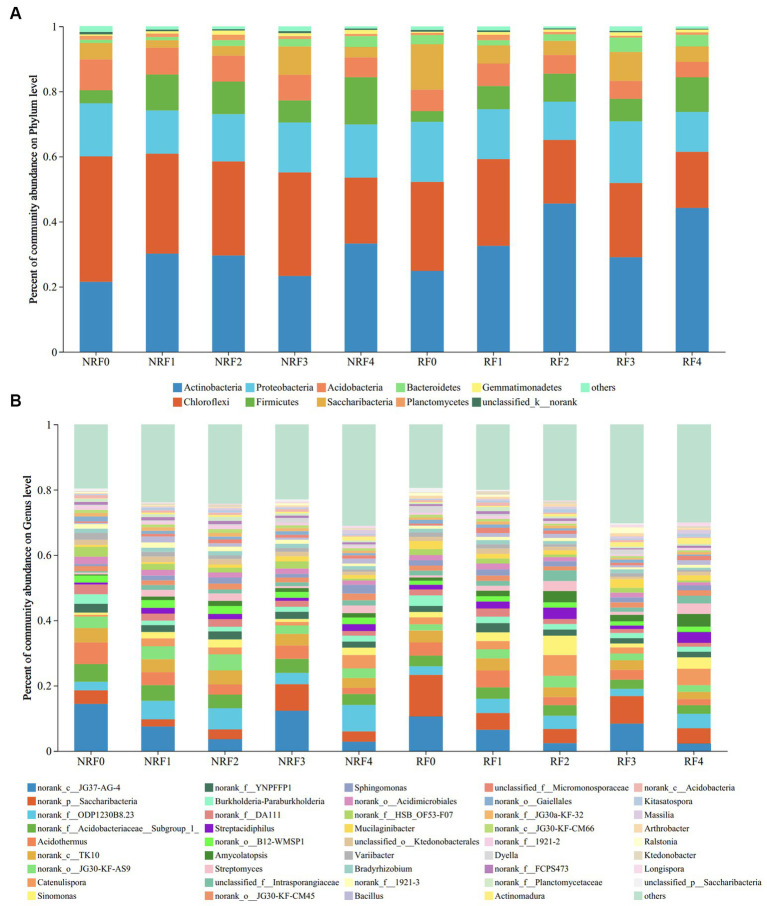
Community composition at the phylum level **(A)** and Genus level **(B)**.

*Actinobacteria*, *Proteobacteria*, and *Chloroflexi* dominated both rhizosphere and non-rhizosphere soils, with the relative abundance of *Actinobacteria* at 37 and 29%, and *Chloroflexi* at 22 and 29%, respectively. Compared to the F0 treatment receiving only chemical fertilizer, the application of organic fertilizer enhanced the relative abundance of *Actinobacteria* while decreasing that of *Chloroflexi*, in both rhizosphere and non-rhizosphere soils. The abundance of *Proteobacteria* remained relatively stable in all treatments (Figure 2A). At the genus level, the dominant genera observed in the oilseed rape soil were mostly uncultured (norank_c_JG37-AG-4, 2.30–14.39%; norank_p_*Saccharibacteria*, 2.19–12.43%, and norank_f_ODP1230B8.23, 2.25–7.94%). These bacterial genera responded significantly to fertilizer treatments. Apart from the F3 treatment that receiving 75% of organic fertilizer, the relative abundance of Norank_c_JG37-AG-4 group demonstrated a decreasing trend along increasing proportion of applied organic fertilizers. Meanwhile, the relative abundance of norank_p_*Saccharibacteria* and norank_f_ODP1230B8.23 differed in soil compartments, showing dominance in rhizosphere and non-rhizosphere soils, respectively (Figure 2B).

**Figure 3:**
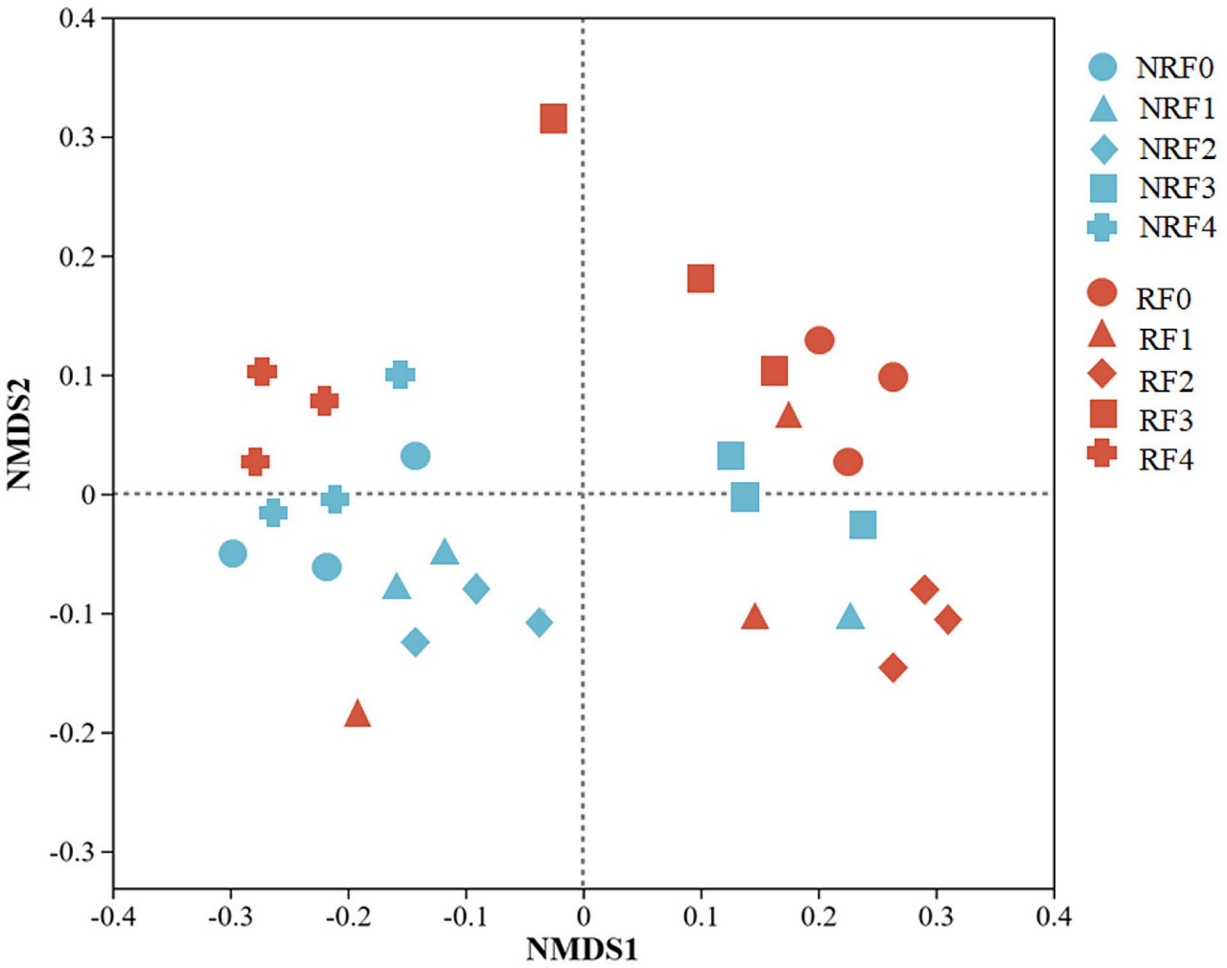
Non-metric Multidimensional Scale ordination of bacterial communities.

NMDS, ANOSIM and PERMANOVA analysis all indicated that the microbial communities differed between rhizosphere and non-rhizosphere soils, as well as among treatments receiving different fertilizer applications (Figure 3, Table 2; Supplementary Table S1).

**Table 2:** Intergroup similarity analysis of rhizosphere effects and fertilization treatments (Adonis).

Adonis	MeanSqs	R^2^	P
REs	0.206	0.087	0.030*
F	0.263	0.545	0.001**

**Figure 4:**
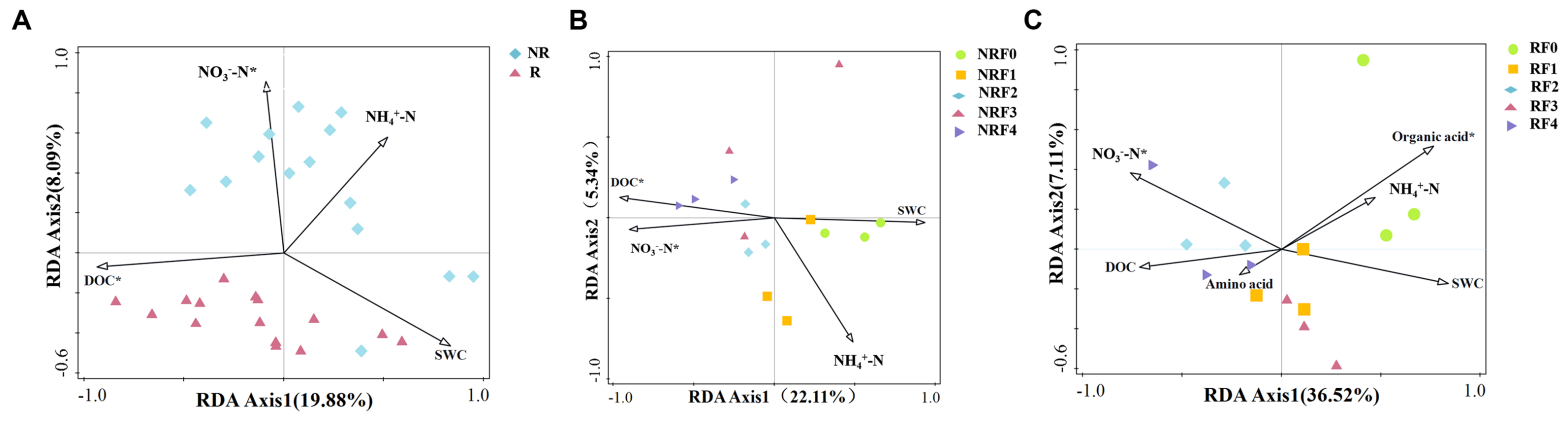
Redundancy analysis of soil bacterial community structure and soil physicochemical properties in oilseed rape with different fertilizer treatments. **(A)** Rhizosphere and non-rhizosphere soil bacterial community structure; **(B)** Rhizosphere fertilization treatment soil bacterial community structure; **(C)** Non-rhizosphere fertilization treatment soil bacterial community structure.

Redundancy analysis (RDA) highlighted the importance of DOC in influencing rhizosphere bacterial community structure, while nitrate nitrogen in shaping non-rhizosphere microbial community structure (Figure 4A). In addition, factors such as organic acids and DOC were also significantly associated with bacterial community structure in the rhizosphere and non-rhizosphere soil fertilization treatments, respectively (Figures 4B,C).

### 3.4 Pathogenic bacteria abundance in organic fertilization treatments

Soil pathogenic bacteria analysis revealed that *Ralstonia solanacearum* (*R. solanacearum*) and *Clavibacter michiganensis subsp. Michiganensis* (Cmm) accounted for a comparatively high relative abundance at the OTU level of 0.95–3.8 and 1.00–5.75%, respectively. And their relative abundance was generally higher in the rhizosphere than in non-rhizosphere soils (Table 3). Other pathogenic bacteria *Xanthomonas arboricola pv. Juglandis* (*X. juglandis*) and *Pseudomonas syringae* (Pst) had a lower relative abundance of less than 1% (Table 3). The highest abundance of *R. solanacearum* was found in both rhizosphere and non-rhizosphere soils of F0 treatment receiving chemical fertilizer alone, and organic fertilizer application reduced its abundance. In contrast, the relative abundance of Cmm was enhanced in treatments with organic fertilizers, and this phenomenon was more evident in non-rhizosphere soils. The rest pathogens with lower relative abundance were also slightly enriched in treatments receiving combined organic-chemical fertilizers than chemical fertilizer alone.

**Table 3:** The relative abundance of pathogenic bacteria in soil samples.

		*R. solanacearum*	Cmm	*A. tumefaciens*	*X. juglandis*	Pst
NR	F0	3.26 ± 1.14a	1.00 ± 0.11c	0.08 ± 0.02c	0.00 ± 0.00c	0.00 ± 0.00c
	F1	1.01 ± 0.20b	4.03 ± 1.04a	0.08 ± 0.03c	0.023 ± 0.01bc	0.01 ± 0.01bc
	F2	0.95 ± 0.12b	2.21 ± 0.20b	0.14 ± 0.05ab	0.04 ± 0.03b	0.02 ± 0.01ab
	F3	1.88 ± 0.35b	2.94 ± 0.71b	0.17 ± 0.06ab	0.05 ± 0.04b	0.01 ± 0.00b
	F4	1.16 ± 0.56b	2.78 ± 0.27b	0.18 ± 0.06a	0.17 ± 0.01a	0.02 ± 0.00a
R	F0	3.80 ± 1.33a	3.75 ± 0.36b	0.23 ± 0.08b	0.00 ± 0.00c	0.01 ± 0.00bc
	F1	2.20 ± 1.17ab	3.59 ± 0.30b	0.1 ± 0.05c	0.01 ± 0.01c	0.00 ± 0.00c
	F2	1.01 ± 0.09b	5.75 ± 0.89a	0.14 ± 0.01bc	0.04 ± 0.01bc	0.02 ± 0.00ab
	F3	2.90 ± 1.09ab	3.27 ± 0.51b	0.56 ± 0.08a	0.12 ± 0.12ab	0.02 ± 0.01a
	F4	1.00 ± 0.41b	4.86 ± 0.77a	0.19 ± 0.03bc	0.16 ± 0.03a	0.01 ± 0.01ab

### 3.5 Co-occurrence network analysis of soil bacterial communities

**Figure 5:**
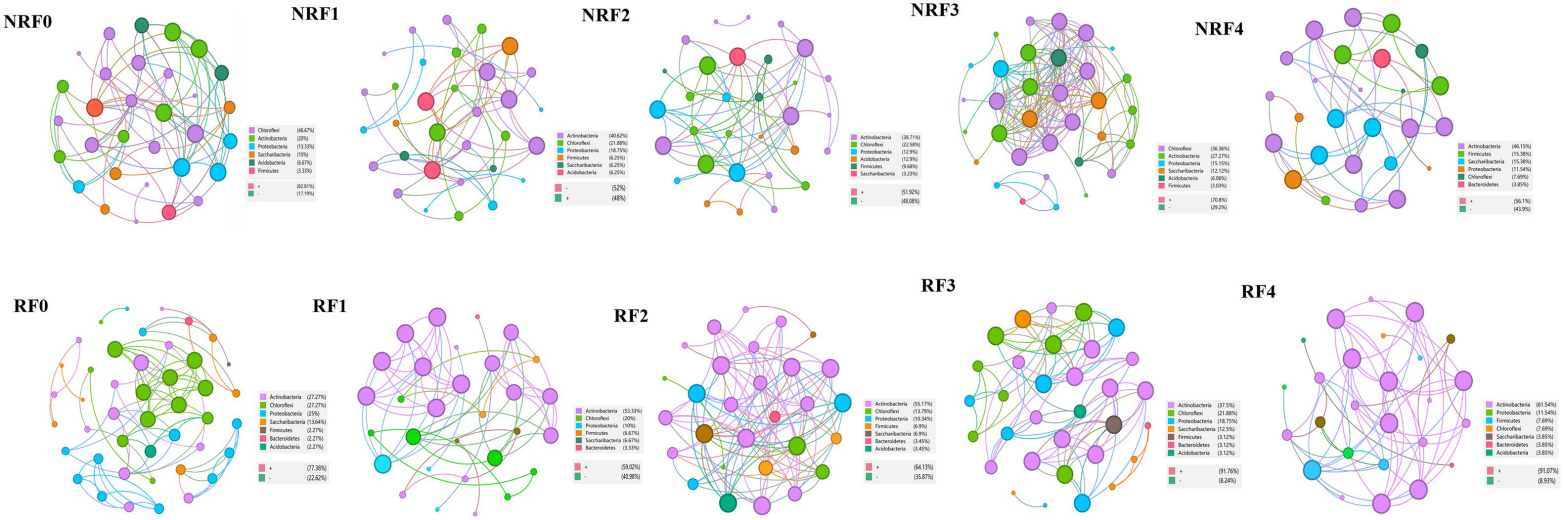
Co-occurrence network analysis based on Spearman’s correlation analysis for connectivity and module partitioning based on selected OTUs. (R,NR) Rhizosphere and non-rhizosphere; (NRF) Non-rhizosphere soil fertilization treatment; (RF) Rhizosphere soil fertilization treatment.

Fertilization and rhizosphere effects influenced microbial networks (Supplementary Table S2). *Actinobacteria* and *Chloroflexi* were the keystone taxa comprising most of the highly connected nodes in both rhizosphere and non-rhizosphere microbial networks, neither of which, however, formed clear modules. There were more positive edges in the rhizosphere network. In the rhizosphere microbial networks of different fertilization treatments, keystone taxa shifted from *Chloroflexi* in the F0 treatment to *Actinobacteria* in treatments with organic fertilizers (Figure 5). The F3 treatment, which received 75% of organic fertilizer, had highly connected nodes from more taxa, including *Chloroflexi*, *Actinobacteria*, and *Proteobacteria*, and exhibited a trend of modularity (Figure 5, RF3). Positive interactions were predominant, especially in the treatments with higher proportions of organic fertilizers. In non-rhizosphere microbial networks, there were generally more diverse keystone taxa in all treatments, but no clear modules were formed (Figure 5, NRF0-NRF4).

## 4 Discussion

Organic fertilizer applications provide sustained nutrient supply, enrich soil structure, and promote long-term soil health (Liu et al., 2021). But nutrient contents of organic fertilizers vary widely and their release is often slow. Additionally, animal manure or compost-derived organic fertilizers may carry pathogens and contaminants like antibiotic resistance genes, bringing concerns for their application (Xu et al., 2020; Wei et al., 2022). So, as an integrated nutrient management practice, the combined application of chemical and organic fertilizers offers several benefits, such as providing a balanced nutrient supply and mitigating the environmental risks associated with the excessive use of synthetic fertilizers (Liu et al., 2022; Wu et al., 2022). However, balancing the application rates of chemical and organic fertilizers and determining the optimal ratio and timing of application can be challenging. Soil microbial diversity and functionality can be a potential indictor.

Some short-term field studies have reported reduced soil bacterial diversity with organic fertilizer application (Tian et al., 2015), others emphasize the enriching potential of organic fertilizers, attributing to their nutrient-rich content, thus promoting bacterial growth and increasing bacterial population and diversity (Ji et al., 2018; Feng et al., 2021). However, the optimal fertilizer rate is still controversial and may be potentially influenced by varying soil types and crops. Application of 10–30% organic fertilizer significantly increased bacterial diversity in a maize pot experiment by Han et al. (2021). In the current study, the most pronounced effect was observed with the application of 75% organic fertilizer, consistent with some previous studies (Ren et al., 2021; Yang et al., 2023). Additionally, the dry weight of the aboveground oilseed rape biomass was the highest in the F3 treatment, indicating a better growth of oilseed rape under 75% of organic fertilization (Yang, 2019). Normally, the application of different rates of organic and chemical fertilizers coordinates the availability of different nutrients (Bao et al., 2020), and can lead to distinct responses of microbial populations (Lin et al., 2019), consequently, the effect of fertilizer application on microbial community assemblage and plant growth differs.

Fertilizer application changes environmental factors including contents of ammonium nitrogen, nitrate nitrogen, and DOC, therefore significantly affecting bacterial abundance and diversity. *Actinobacteria* abundance increased after the application of organic fertilizer. In contrast, the relative abundance of *Chloroflexi* was higher at F0. Studies have reported that *Chloroflexi* is oligotrophic, adapted to nutrient-limited environments, and sensitive to changes in nitrogen and carbon levels (Trivedi et al., 2016; Dai et al., 2019; Romero-Salas et al., 2021). In contrast, *Actinobacteria* can rapidly decompose organic matter, which increases levels of carbon and other nutrients to improve the soil environment. Thus its relative abundance increases with the application of organic fertilizers (Yan et al., 2021). This selective effect helps to maintain the balance of soil ecosystems, but may also trigger certain microbial diseases.

*Ralstonia solanacearum* can cause plant greening and is one of the most common soil-borne diseases. The application of organic manure from swine manure alters the composition of the soil microbial community and reduces the community of *Ralstonia solanacearum* (Gorissen et al., 2004). However, it is widely known that organic fertilizer application may introduce exogenous pathogens. Potential pathogenic bacteria such as *Pseudomonas*, *Flavobacterium*, and *Bacillus* were detected in more than 13% of organic fertilizer products (Xu et al., 2022). Our results showed that the combined application of organic manure with chemical fertilizers significantly suppressed *R. solanacearum* compared to chemical fertilizers alone. In addition, Cmm was similarly abundant in F0 treatment and other treatment receiving organic fertilizers, and other pathogens did not show increasing abundance with increasing proportion of organic fertilizer applied (Table 3), suggesting that these pathogens were not introduced solely, if not at all, by organic fertilizer application in this study.

Although many studies have shown that organic fertilizers inhibit a wide range of plant soil-borne diseases, soil diseases are usually caused by multiple soil pathogens, and it is difficult to effectively inhibit multiple pathogens by applying a single organic fertilizer alone; therefore, there is a need for more in-depth research on the application of fertilizer mixtures, such as the mixing of multiple organic fertilizers, organic fertilizers, and biocontrol formulations, as well as mixing of organic fertilizers with induced activators (Liu et al., 2017; Yang et al., 2022). In addition to this, the application of organic fertilizers after soil fumigation can activate beneficial soil microorganisms, promote soil health, and increase crop yield (Li Q. et al., 2022).

The microbial networks of F2 and F3 treatments receiving 50 and 75% of organic fertilizers application had the highest number of nods and edges in the rhizosphere and non-rhizosphere soils, respectively, suggesting high complexity and stability of these microbial communities (Okuyama and Holland, 2007). Complex networks often support more diverse functions, resulting in increased efficiency of resource cycling and information transfer (Morriën et al., 2017; Wagg et al., 2019), which also improve the stability of microbial communities (Wang et al., 2017; Ji et al., 2020). Positive and negative associations, which indicate cooperation and competition among species, respectively, are key network attributes (Deng et al., 2015). It has been suggested that a high positive correlation may lead to dependence and mutual collapse (Coyte et al., 2015), and conversely, the negative correlation may stabilize co-oscillations in microbial communities and enhance network stability (Zhou et al., 2020). Yang et al. found a higher negative correlation in microbial networks under the combination of organic and inorganic fertilizers, which suggests that the combined application of fertilizers resulted in microbial communities with higher network stability (Yang et al., 2023). However, it should be noted that co-occurrence networks infer biological interactions based on co-occurrences or absence of sequences, it is important to exercise caution when drawing conclusions from microbial networks (Faust, 2021).

Studies have reported that plant roots impact the soil microbial community by releasing a substantial amount of exudates, including primary metabolites such as amino acids, organic acids, sugars, and carboxylic acids (Li et al., 2018). This release enhances the carbon and nitrogen substrate nutrients for microbial growth, turning the rhizosphere into a favorable microhabitat for the rapid colonization of specific microorganisms (Ulbrich et al., 2022; Steinauer et al., 2023). Simultaneously, these secretions exhibit selectivity in promoting the growth and colonization of particular soil microbes with certain potential metabolic profiles (Bulgarelli et al., 2013; Li et al., 2020; He et al., 2022). Plants recruit beneficial microbial groups through rhizospheric exudates, increasing rhizobacterial abundance and a decrease in diversity (Ling et al., 2022), aligning with our research findings that rhizosphere effects significantly influenced the rhizosphere microbial diversity and abundance.

Research indicates that specific plant metabolites, such as organic acids, amino acids, and hormones, can serve as signaling molecules, activators, and attractants, influencing the composition of microbial communities (Baetz and Martinoia, 2014). Many studies have shown that different rhizosphere secretions attract different microbial communities. For instance, organic acid secretion was found to attract *Comamonadacea* against pathogenic bacteria; Arabidopsis thaliana attracts and stimulates specific *Pseudomonas* colonies by releasing long-chain amino acids, etc. (Yuan et al., 2018; Wen et al., 2020). Our study also observed significant differences between rhizosphere and non-rhizosphere soil microbial communities, and organic acids was recognized as a pivotal driver in the formation of these differences. Together, these studies highlight the intricate interactions among fertilization, crop root exudates and soil microbial communities.

The complexity and negative correlations of rhizosphere microbial networks are lower than those in non-rhizosphere environments, which could be attributed to the loss of diversity and enriching effects of rhizosphere secretions (Chen Y. et al., 2023), driven by changes in rhizosphere resource availability and niche differentiation (Zhao et al., 2020). Currently, many studies are exploring applications of rhizosphere effect in plant restoration. For instance, the halophyte L. sinense under salt stress can attract and recruit beneficial rhizosphere bacteria through root exudates, and the recruited B. flexus KLBMP 4941 enhances seedling salt tolerance through intricate plant physiological regulatory mechanisms (Xiong et al., 2020). Similarly, Luo et al. investigated the succession trajectories and oscillations of rhizosphere microbial symbiotic networks in S. alfredii, aiming to deepen understanding and design stable bioremediation microbial communities (Luo et al., 2021). Understanding the interactions and feedback loop involving fertilization, crop root exudates, the soil microbial community and soil properties, offers a new avenue for sustainable agricultural production.

## 5 Conclusion

The study demonstrates that the application of combined organic fertilizer and chemical fertilizer, together with rhizosphere effects, fosters a more stable and conducive soil bacterial community, and enhances oilseed rape growth. Nonetheless, the research primarily focused on the seedling stage, prolonged investigations will be necessary to understand the comprehensive impacts of fertilization on bacterial community structure and function, and reveal the best ratio of organic to chemical fertilizers in oilseed rape cultivation.

## Data availability statement

The datasets presented in this study can be found in online repositories. The names of the repository/repositories and accession number(s) can be found in the article/Supplementary material.

## Author contributions

JW: Conceptualization, Data curation, Investigation, Validation, Visualization, Writing – original draft, Writing – review & editing. HQ: Funding acquisition, Resources, Supervision, Writing – review & editing. LZ: Investigation, Supervision, Validation, Writing – review & editing. YT: Investigation, Supervision, Writing – review & editing. JL: Conceptualization, Investigation, Visualization, Writing – review & editing. HX: Investigation, Methodology, Supervision, Validation, Writing – review & editing. BZ: Conceptualization, Data curation, Funding acquisition, Resources, Supervision, Validation, Visualization, Writing – review & editing.
