# Supplementary material for: Synergistic effects of rhizosphere effect and combined organic and chemical fertilizers application on soil bacterial diversity and community structure in oilseed rape cultivation
Source: Front Microbiol. 2024 Mar 14;15:1374199. doi: 10.3389/fmicb.2024.1374199 (PMC10972979; doi:10.3389/fmicb.2024.1374199)
Supplement: Supplementary file 1 [file Data_Sheet_1.docx]

Supplementary Material

**Synergistic Effects of Rhizosphere Effect and Combined Organic and Chemical Fertilizers Application on Soil Bacterial Diversity and Community Structure in Oilseed Rape Cultivation**

**Jingyuan Wang1,2, Hongling Qin2, Leyan Zhang1,2, Yafang Tang3, Junjiang Long1*, Huaqin Xu1, Baoli Zhu2***

**1. College of Resources and Environment, Hunan Agricultural University, Changsha 410128, China**

**2. Key Laboratory of Agro-ecological Processes in Subtropical Region, Institute of Subtropical Agriculture, Chinese Academy of Sciences, Changsha 410125, China**

**3. Hubei Key Laboratory of Quality Control of Characteristic Fruits and Vegetables, College of Life Science and Technology, Hubei Engineering University, Xiaogan 432000, China**

*** Correspondence: J. Long, long0109_agro@163.com; and B. Zhu, baoli.zhu@isa.ac.cn**

**Table S1** Comparison between different treatments based on bacterial community

|  | ANOSIM | | PERMANOVA | |
| --- | --- | --- | --- | --- |
| Group | R | P | R^2^ | P |
| R/NR | 0.119 | 0.038* | 0.086 | 0.036* |
| NRF | 0.7119 | 0.001** | 0.587 | 0.002** |
| RF | 0.470 | 0.003** | 0.589 | 0.001** |

**Table S2** Indicators for network analysis based on bacterial communities

| Group | nods | edges | graph density | average degree | modularity |
| --- | --- | --- | --- | --- | --- |
| NRF0 | 31 | 64 | 0.138 | 4.129 | 0.779 |
| NRF1 | 32 | 50 | 0.101 | 3.125 | 0.769 |
| NRF2 | 31 | 52 | 0.112 | 3.355 | 0.672 |
| NRF3 | 33 | 137 | 0.259 | 8.303 | 0.387 |
| NRF4 | 26 | 41 | 0.126 | 3.154 | 0.794 |
| RF0 | 44 | 84 | 0.089 | 3.818 | 0.815 |
| RF1 | 30 | 61 | 0.14 | 4.067 | 0.663 |
| RF2 | 29 | 92 | 0.227 | 6.345 | 0.640 |
| RF3 | 32 | 85 | 0.171 | 5.312 | 0.737 |
| RF4 | 26 | 56 | 0.172 | 4.308 | 0.542 |


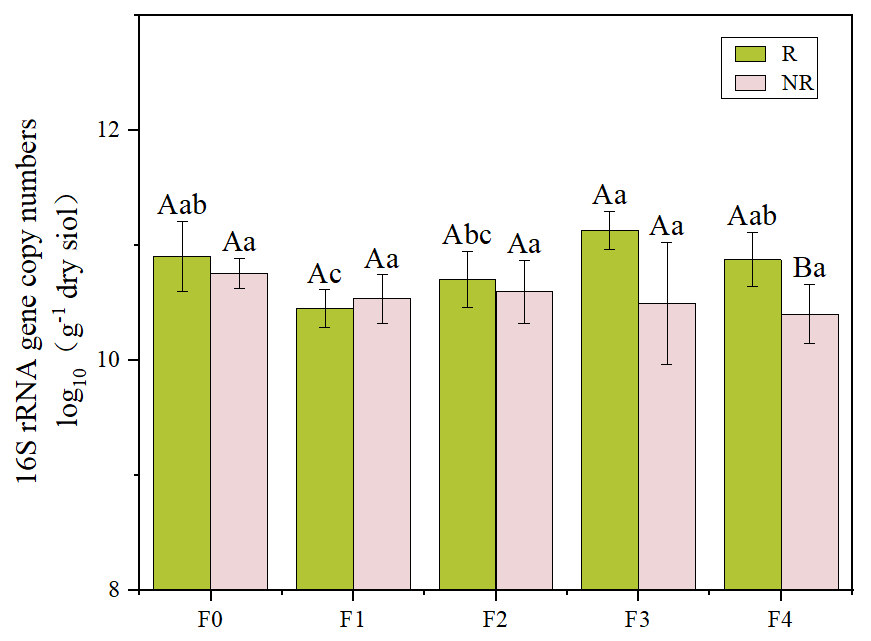


**Figure S1**

The abundance of 16S rRNA genes in soil bacteria from rhizosphere and non-rhizosphere soil under different fertilization treatments. Uppercase letters indicate differences between rhizosphere and non-rhizosphere between each group of fertilizer treatments；lowercase letters indicate differences between fertilizer treatments (p<0.05)

**
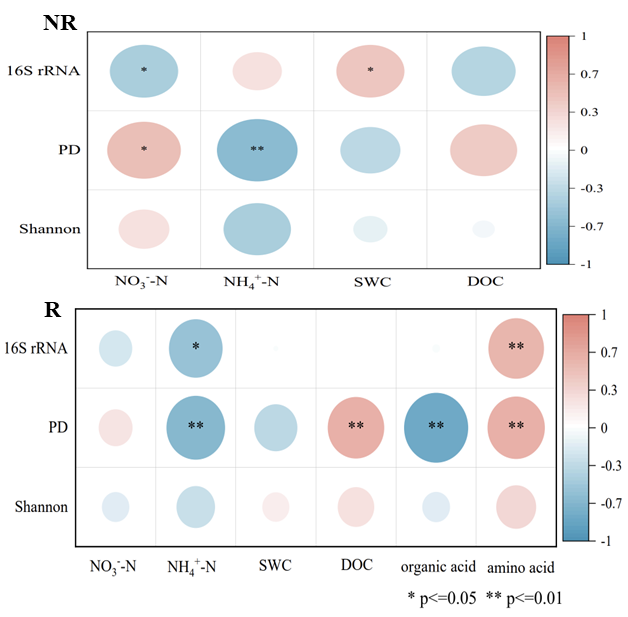
**

**Figure S2**

Correlation of soil physicochemical properties with bacterial abundance and species α diversity
